# Roles of Proteoglycans and Glycosaminoglycans in Wound Healing and Fibrosis

**DOI:** 10.1155/2015/834893

**Published:** 2015-09-10

**Authors:** Shibnath Ghatak, Edward V. Maytin, Judith A. Mack, Vincent C. Hascall, Ilia Atanelishvili, Ricardo Moreno Rodriguez, Roger R. Markwald, Suniti Misra

**Affiliations:** ^1^Department of Regenerative Medicine and Cell Biology, Medical University of South Carolina, Charleston, SC 29425, USA; ^2^Department of Biomedical Engineering, Cleveland Clinic, Cleveland, OH 44195, USA; ^3^Division of Rheumatology & Immunology, Department of Medicine, Medical University of South Carolina, 114 Doughty Street, Charleston, SC 29425, USA

## Abstract

A wound is a type of injury that damages living tissues. In this review, we will be referring mainly to healing responses in the organs including skin and the lungs. *Fibrosis* is a process of dysregulated extracellular matrix (ECM) production that leads to a dense and functionally abnormal connective tissue compartment (dermis). In tissues such as the skin, the repair of the dermis after wounding requires not only the *fibroblasts* that produce the ECM molecules, but also the overlying epithelial layer (*keratinocytes*), the *endothelial cells*, and *smooth muscle cells* of the blood vessel and white blood cells such as *neutrophils* and *macrophages*, which together orchestrate the cytokine-mediated signaling and paracrine interactions that are required to regulate the proper extent and timing of the repair process. This review will focus on the importance of extracellular molecules in the microenvironment, primarily the proteoglycans and glycosaminoglycan hyaluronan, and their roles in wound healing. First, we will briefly summarize the physiological, cellular, and biochemical elements of wound healing, including the importance of cytokine cross-talk between cell types. Second, we will discuss the role of proteoglycans and hyaluronan in regulating these processes. Finally, approaches that utilize these concepts as potential therapies for fibrosis are discussed.

## 1. Introduction

Our understanding of the biology of wound healing has advanced significantly in recent years. A major goal is to determine what are the biochemical/physiological factors in the wound that can reconstruct the damaged parts more effectively. Wound healing is a dynamic interactive process involving many precisely interrelated phases that overlap in time and lead to the restitution of tissue integrity. The healing process reflects the complex and coordinated body response to tissue injury resulting from the interactions of different cell types and extracellular matrix components. Failure of coordinated regulation can result in tissue fibrosis with excessive collagen production and, if highly progressive, the fibrotic process may eventually lead to organ malfunction and death. Most chronic wounds are associated with fibrosis of various organs, ischemia, or diabetes mellitus and affect from 3 to 6 million people in the USA, with older persons (>65) accounting for 85% of these events. Nonhealing wounds result in enormous health care expenditures, with the total cost estimated to be more than $3 billion* per *year [1].

The importance of the ECM in the complex processes of wound healing is that it provides architectural support for the tissues and a platform for cells and molecules that regulate inter- and intracellular signaling. ECMs are secreted molecules that constitute the cell microenvironment and are composed of a dynamic and complex array of glycoproteins, collagens, glycosaminoglycans (GAGs), and proteoglycans (PGs). Among these, the GAG hyaluronan (HA) and the PGs such as versican and aggrecan are all partners in the control of the wound healing process. It is now well accepted that the ECM not only provides architectural support for resting tissues, but also undergoes important alterations after injury that are essential for directing cell behavior during the wound healing process. The function of the ECM facilitates repair of the wound either directly by modulating important aspects of cell behavior such as adhesion, migration, proliferation, metabolism, differentiation, and survival, or indirectly by modulating extracellular protease secretion/activation, or by modulating growth factor activity or bioavailability. Cells have specific transmembrane receptors that recognize ECM components and interact with the intracellular cytoskeleton and signaling pathways [2]. Classic examples of ECM interactions with cells that fulfill the criteria of anchoring and adhesion to receptors that modulate intracellular signaling pathways involve cell surface receptors such as integrins and the HA receptor CD44 [3–5]. Receptors on ECM are involved in many pathological processes, including inflammation, fibrosis diseases, and cancer [6–8]. Although it is clear that a cascade of ECM molecules, including GAGs, PGs, connective tissue glycoproteins, and cell surface adhesion receptors, are involved in wound healing, we will primarily address the problem of wound healing with abnormal fibrosis by focusing on the role of the cell-adhesion molecule CD44 and its principal ligand HA in wound healing and tissue fibrosis.

## 2. Biochemical and Physiological Characteristics of Wound Healing

### 2.1. Wound Healing and Fibrosis

Wounds are injuries to a living tissue. The cellular, molecular, biochemical, and physiological events associated with wound healing permit living tissue to repair tissue injury. This process consists of a highly orchestrated sequence of events that require the collaborative efforts of many different cell types, including blood cells, epithelial and connective tissue cells, inflammatory cells, and many soluble factors, such as coagulation factors, growth factors, and cytokines. The behaviour of each of the participating cell types during the phases of proliferation, migration, matrix synthesis, and contraction, as well as the soluble factor and matrix signals present at a wound site, is crucial for repairing the tissue injury. It is a dynamic and strongly regulated process that starts immediately after the initial lesion, and it will last until complete closure of the wound and regeneration of the tissue as functional as possible occurs. Fibroblasts are the principal biosynthetic cells producing interstitial collagens, fibronectins, and other matrix components. They also differentiate into myofibroblasts, a specialized contractile cell type responsible for closure of the wound. In the setting of repetitive trauma or certain pathological states, increased ECM deposition of abnormal matrix (scarring; fibrosis) occurs in a variety of fibrotic diseases in tissues, including liver [9], kidney [10], lung [11], and heart [12, 13], and in scleroderma [14, 15]. Collagen deposition in the matrix is a requisite and, typically, reversible part of wound healing. However, in fibrosis, normal tissue repair can evolve into a progressively irreversible fibrotic response with fibroblast differentiation to excessive numbers of myofibroblasts and increased collagen deposition.

### 2.2. Sequence of Processes in Wound Healing

**Figure 1:**
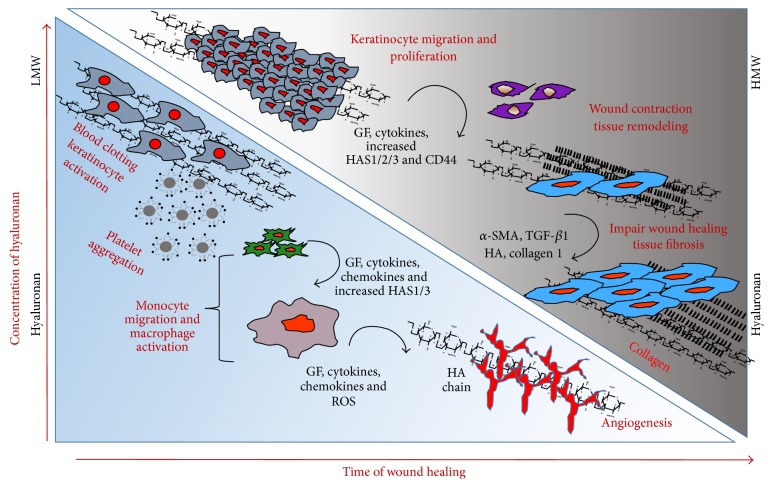
Schematic presentation of changes in hyaluronan synthesis/molecular size and cellular events and matrix events during the course of wound healing and fibrosis. Many of the biological processes mediated by HA are crucial for wound healing and fibrosis. After injury, wound healing follows a tightly regulated sequence of events. These phases are inflammation, granulation tissue formation, proliferation, reepithelization, and remodelling. In the early phases, high molecular HA is degraded by reactive oxygen species from activated granulocytes and by hyaluronidases secreted from platelets. Then monocytes secrete inflammatory mediators, which attract additional inflammatory cells. Keratinocytes become activated to migrate, proliferate, and to synthesize HA. As a result the LMW degradation products are active inducers of angiogenesis and inflammation. At later stages the interim matrix becomes supplemented with newly synthesized HMW HA, which contributes to tissue remodelling. During repetitive injury, the repairing processes are hindered, and the* keratinocytes*, the endothelial cells, and* smooth muscle cells* of the blood vessel,* neutrophils*, and* macrophages* together orchestrate the increased cytokine-mediated signaling and augment HA-CD44 signaling and excess collagen production that results in fibrosis.

Wound healing involves integrated and overlapping phases: (a) haemostasis, (b) inflammation, (c) proliferation, and (d) remodelling (Figure 1).

#### 2.2.1. The Homeostasis of Wound Healing

Immediately after the injury, vascular constriction and platelet aggregation at the injury site form a fibrin clot, which reduces leakage of blood from damaged blood vessels in the wound. The fibrin clot is a temporary shield containing many important molecules: fibronectin (FN), SPARC (Secreted Protein, Acidic and Rich in Cysteine), thrombospondin, vitronectin, and growth factors such as transforming growth factor-*β* (TGF-*β*), platelet-derived growth factor (PDGF), fibroblast growth factor (FGF), epidermal growth factor (EGF), and insulin-like growth factor-1 (IGF-1) released by platelets and monocytes [16]. Components of the fibrin clot also bind to cells and to other ECM proteins simultaneously [17]. The clot then provides a provisional matrix for migration of the cells to pass over and through during the wound repair process [16, 18].

#### 2.2.2. The Inflammation Phase

Once the bleeding is controlled, sequential infiltration of inflammatory cells, such as neutrophils, macrophages, and lymphocytes into the wound (chemotaxis) promote the inflammatory phase [19–21]. A critical function of neutrophils is the clearance of invading microbes and cellular debris in the wound area, although these cells also produce substances such as proteases and reactive oxygen species (ROS), which can cause additional damage. Unless a wound is grossly infected, the neutrophil infiltration terminates within a few days, and expended neutrophils will be phagocytosed by tissue macrophages, which then degrade nonviable tissue and dead bacteria. Inflammation lasts as long as there is debris in the wound. However, inflammation can lead to the damage of tissue if it lasts too long. Thus, the reduction of inflammation is frequently a goal in therapeutic settings.

#### 2.2.3. The Proliferation and Migratory Phase

By clearing the apoptotic cells, macrophages help the resolution of inflammation, and they undergo a phenotypic transition to a reparative state that stimulates keratinocytes, fibroblasts, and angiogenesis to promote tissue regeneration [22, 23]. T-lymphocytes migrate into wounds following the inflammatory cells and macrophages, and they peak during the late-proliferative/early-remodelling phase. Although the role of T-lymphocytes is not completely understood, studies have reported that CD4+ cells (T-helper cells) have a stimulatory role while CD8+ cells (T-suppressor-cytotoxic cells) have an inhibitory role in wound healing [24, 25]. Blood factors are released into the wound that cause the migration and division of cells, which prepares them for the proliferative phase. In this way, macrophages promote the transition to the proliferative phase of healing.

#### 2.2.4. The Reparative Phase and Remodeling

The* reparative phase and remodeling* is characterized by the formation of the granulation tissue that fills the wound before reepithelialization where epithelial cells migrate across the new tissue to form a barrier between the wound and the environment. Granulation tissue contains fibroblasts and endothelial cells in an ECM that contains GAGs and PGs [26], which supports capillary growth, fibronectin, and collagen formation at the site of injury so that vascular density of the wound can return to normal. Thus, following robust proliferation and ECM synthesis, wound healing enters the final remodelling phase, where the wound also undergoes physical contraction mediated by contractile fibroblasts (myofibroblasts) that appear in the wound [20, 21] (Figure 1).

## 3. Modulators of Fibrosis in Wound Healing

### 3.1. Soluble Mediators in the ECM during Wound Healing and Fibrosis

The time-dependent sequence of events in wound healing includes regulation of cell-ECM interactions that are controlled by soluble mediators that act synergistically to direct wound remodelling by regulating ECM synthesis and degradation. Subsequently, the myofibroblast population is also expanded as a result of epithelial cells undergoing epithelial-to-mesenchymal transition (EMT) and of the activation of resident fibroblasts that leads to ECM deposition and tissue remodeling. The types of soluble mediators released during tissue injury are described below.

Following tissue injury, platelets aggregate and release platelet-derived growth factor-AB (PDGF-AB) from the granules. Consequent infiltration of macrophages provides an additional source of PDGF-AB. PDGFs are potent mitogens and chemoattractants for many cells, including fibroblasts, smooth muscle cells, mesenchymal cells, neutrophils, and monocytes, and they upregulate fibronectin, procollagen, and collagen activities. PDGFs have crucial roles in fibrotic disorders such as kidney, lung, and skin fibrosis [10, 27–29]. Healing of the wounds involves increased infiltration of inflammatory cells and fibroblasts followed by a marked increase in collagen deposition at the wound site. TGF-*β*1 influences collagen degradation by stimulating tissue inhibitor of metalloproteinase (TIMP), which inhibits protease activity and decreases degradation of newly synthesized collagen [30–33]. We and others showed that blocking TGF-*β*1 decreases ECM deposition, scar formation, and fibrosis [14, 34]. Like PDGF, the fibrogenic potential of TGF-*β*1 makes it a prime candidate for drug therapy in settings of tissue fibrosis [35].

FGFs are strongly mitogenic for endothelial cells and are involved in angiogenesis, directing endothelial cell migration, proliferation, and plasminogen activator synthesis [36]. IGFs are produced by several cell types including macrophages and fibroblasts [37, 38], and they have the potential to activate fibroblasts by either stimulating replication or increasing the production of connective tissue components such as collagen, elastin, and PGs, including versican [39, 40]. EGF acts as a mitogenic factor for cells including fibroblasts, keratinocytes, smooth muscle cells, and epithelial cells [41–44] and increases skin wounds [45]. However, exaggeration of this process of repair and the subsequent increased reorganization of the tissue matrix can lead to the development of fibrotic scar tissue that is characterized by excessive accumulation of ECM components, including fibronectin, PGs, HA, and interstitial collagens.

### 3.2. Proteoglycans in Wound Healing

**Figure 2:**
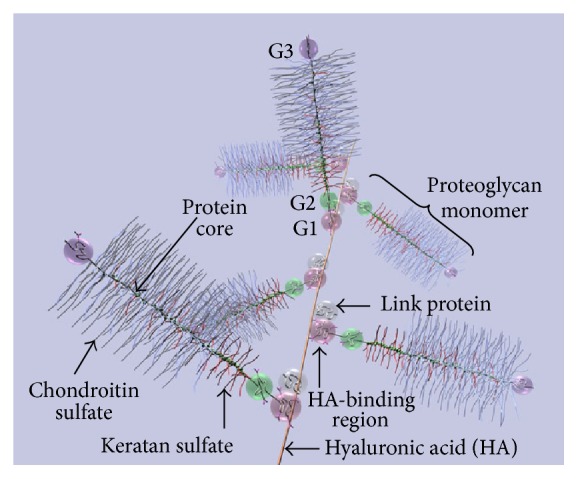
Diagram of part of an aggrecan aggregate. G1, G2, and G3 are globular, folded regions of the central core protein. Proteoglycan aggrecan showing the noncovalent binding of proteoglycan to HA with the link proteins.

PGs have core proteins or glycoproteins with large GAG side-chains (Figure 2), and they participate in cell-cell and cell-matrix interactions, cell proliferation, and migration, and in cytokine and growth factor signaling associated with wound healing. Small leucine-rich PGs (SLRPs) and the chondroitin sulfate PG versican are found in the dermis of wounds, the PG perlecan in the basement membrane, and the heparan sulfate PGs, syndecans, and glypicans on the cell surfaces. The versican-v3 isoform promotes transition of normal dermal fibroblasts to myofibroblasts [46, 47]. Perlecan regulates wound healing through induction of angiogenesis [48]. Increased expression of syndecans-1 and syndecans-4 in wounds [49] stimulates keratinocyte [50] and endothelial cell migration and angiogenesis in mice [51]. Decorin, a member of the SLRP family, negatively regulates TGF-*β*1 [52] and demonstrates effects of antifibrosis in various tissues, including kidney [53], muscle [54], and lung [55]. GAG chains covalently bound to the core protein of PGs dominate their physical properties. PGs can maintain the ECM in a hydrated condition, exclude other macromolecules, and allow permeability of low molecular weight solutes. Thus, by interacting with other ECM components, PGs are critical to organize the matrix [56, 57].

### 3.3. Glycosaminoglycans in Wound Healing

**Figure 3:**
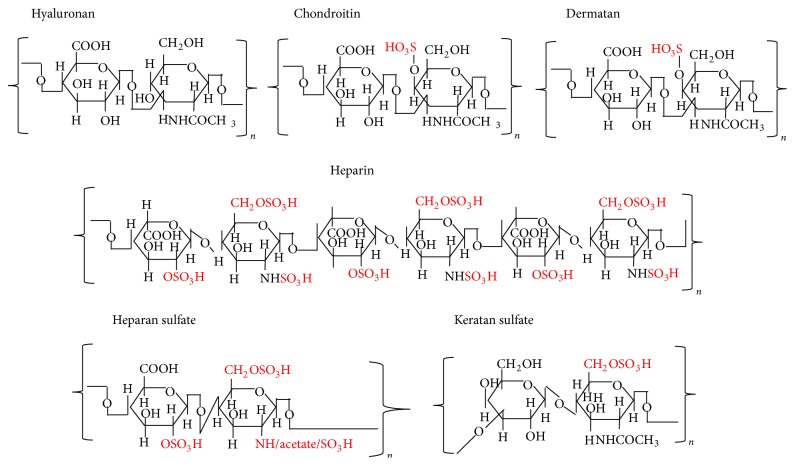
Structures of repeating disaccharides of glycosaminoglycans.

Of the various ECM macromolecules, the GAG side-chains of PGs are very important players in wound healing. GAG chains (Figure 3) exhibit considerable structural diversity resulting from a complex biosynthesis that is tightly regulated in biological systems, enabling the modified GAGs to selectively interact with a variety of ligands in a spatially and temporally controlled manner [56, 57]. During the proliferation phase of wound healing, fibroblasts and other mesenchymal cells enter the inflammatory site of the wound in response to growth factors that are necessary for stimulation of cell proliferation [58]. The fibroblasts synthesize collagen and PGs, which continues for several weeks with proportional increases of collagen. During this time, endothelial cells form capillaries, and the GAGs (HA, chondroitin sulfate (CS), and dermatan sulfate (DS)) also change in their levels. Initially, HA is synthesized in large amounts by the fibroblasts for 2 weeks [26], followed by increased levels of DS and CS PGs [59]. Gradually, when the proliferation of cells reaches a plateau, heparan sulfate (HS) PGs are elevated in the wound. Sulfated PGs with CS and DS assist in collagen polymerization [60], and HS PGs on cells can create anchors to surrounding matrix [61]. PG degradation by proteases in the wounds can release GAG-peptide fragments, which may modulate the wound healing process [62]. For instance, CS and DS can regulate growth factor activity and may stimulate nitric oxide production, which, in turn, can modulate angiogenesis, whereas HS can stimulate the release of IL-1, IL-6, PGE2, and TGF-*β* and contribute to the modulation of its proangiogenic effects in the tissues [63, 64]. Studies have demonstrated colocalization of the large CS PG versican with HA in cables in smooth muscle cells [65] and in an epithelial cell system [66]. Of the GAGs, HA has a key role in each phase of wound healing as well as in regulating ECM organization and metabolism [67].

### 3.4. HA in Wound Healing and Fibrosis

#### 3.4.1. Structure of Hyaluronan

HA is omnipresent in the human body and in all vertebrates, occurring in almost all biological fluids and tissues, with the highest amounts in the ECM of soft connective tissues. HA is a linear, naturally occurring, nonsulfated GAG of the ECM (Figure 3). HA has a repeat of disaccharides consisting of D-glucuronic acid and* N*-acetylglucosamine [68–70]. Native HA has a very high molar mass, usually in the order of millions of Daltons, (10^5^ to 10^7^ Da) before being progressively degraded into smaller fragments in the ECM [14, 67, 70, 71]. It possesses interesting viscoelastic properties based on its polymeric and polyelectrolyte characteristics. Despite its relatively simple structure, HA is an extraordinarily versatile GAG and is involved in several key processes, including early EMT in development and morphogenesis, cell signaling, wound repair and regeneration, matrix organization and pathobiology.

#### 3.4.2. HA during Inflammation in Tissue Injury and Fibrosis

A pattern has emerged; following tissue injury, inflammatory cells, keratinocytes, fibroblasts, endothelial cells, and pluripotent stem cells undergo interactions with ECM macromolecules or their fragments to heal the wound. During the inflammatory phase of wound healing, HA accumulates in the wound bed and acts as a regulator of early inflammation. The major functions of HA in this phase are to modulate inflammatory cell and fibroblast cell migration, proinflammatory cytokine synthesis, and the phagocytosis of invading microbes [67]. In this inflammatory phase, HA degradation products (low-MW HA presumably ~2.5 × 10^5^ Da) can promote early inflammation. At sites of inflammation and tissue injury, these low-MW HA fragments that accumulate from degradation of high molecular weight HA can initiate toll-receptor-2 and toll-receptor-4 (TL-R2 and TL-R4) induction of proinflammatory cytokines IL-6, TNF-*α*, and IL-1*β* [72]. These cytokines, in turn, induce HA production* in vitro* by various cell types, including endothelial cells [73], dendritic cells [74], and fibroblasts [75]. The proliferative phase overlaps with the remodeling phase where keratinocytes differentiate to fibroblasts. During these events, the growth factors and cytokines released by the inflammatory cells induce fibroblast and keratinocyte migration and proliferation. Furthermore, the levels of HA synthesized by both fibroblasts and keratinocytes are elevated during reepithelialization where epithelial cells migrate across the new tissue to form a barrier between the wound and the environment [26] (Figure 1).

The levels of HA and its degradation products are abundant in patients with scleroderma fibrosis and in the animal models of bleomycin-induced lung injury [76, 77]. The excessive production of HA is one of the major events in scleroderma fibrosis [78, 79]. Furthermore, increased HA levels are observed in bronchoalveolar lavage (BAL) fluid and/or plasma from patients with pulmonary fibrosis [80], interstitial lung disease [81], and idiopathic pulmonary injury [82]. However, failure to remove HA fragments from the site of tissue injury contributes to the unremitting inflammation and destruction observed in tissue fibrosis [83]. Clearance of HA fragments depends both on its receptor CD44 [84] and on recognition by the host via TL-R2 and TL-R4 [85] (Figure 1).

#### 3.4.3. HA Synthases and Tissue Injury

**Figure 4:**
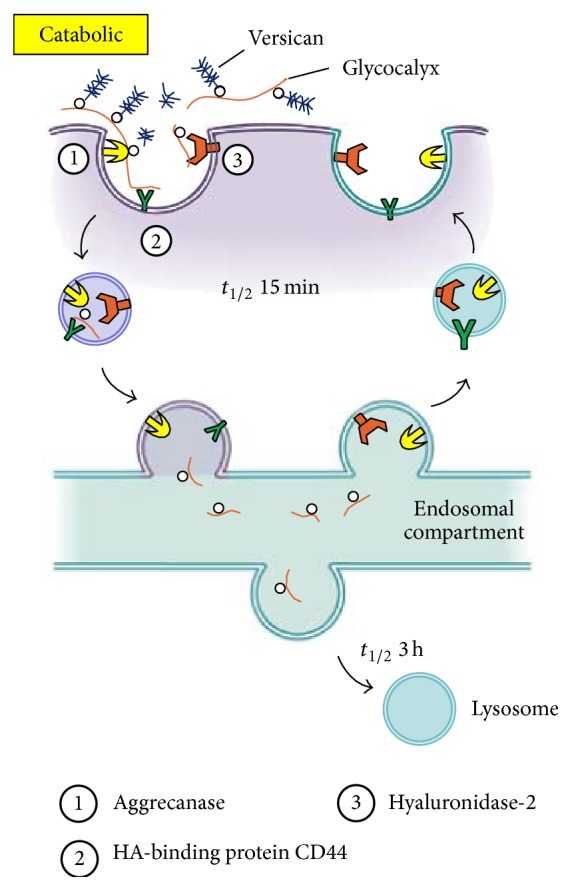
Model for catabolism of pericellular hyaluronan glycocalyx matrices (adapted from [86] with the permission from Dr. Hascall).

Most cells synthesize HA at some point during their life cycles implicating its function for fundamental biological processes. Unlike all of the sulfated GAGs, biosynthesis of HA does not require a core protein and is not done in the cell's Golgi networks. HA is naturally synthesized by a class of integral membrane proteins called HA synthases, of which vertebrates have three types: HAS1, HAS2, and HAS3 [86–88]. The expression of various HAS isozymes is likely to be a fine control system critical for the effective mediation of different cell behaviors. While HAS1 and HAS2 are able to produce large-sized HA (up to 2000 kDa), HA produced by HAS3 is of a lower molecular mass (100–1000 kDa) [89–91]. HAS2 is dynamically regulated at several levels. For example, a number of studies have defined the details of transcriptional regulation of the HAS2 gene promoter in response to a variety of cytokines and growth factors that are released as a result of wounding [92, 93]. Some of the most dramatic effects of cytokines on HA regulation occur in epidermal keratinocytes of the skin, in which HA production is boosted many-fold by exposure to a variety of growth factors including EGFR [94, 95]. Interestingly, wounding of keratinocytes releases HB-EGF, which itself has been shown to upregulate HA synthesis in neighboring cells [96, 97], an example of the paracrine effects (cell-cell cross-talk) that now appear to have a central role in mechanisms of fibrosis (discussed more below). HAS2 activity can also be governed by posttranslational pathways, such as regulation of O-GlcNAcylation. Once in circulation, HA is very effectively removed by hepatic endothelial cells. This efficient process recovers the sugars by internalization and transport to lysosomes [98]. Most cells do not have this option but do have a metabolically active pericellular matrix (glycocalyx). (Figure 4) For example, keratinocytes catabolize hyaluronan by a mechanism that involves the CD44 HA receptor [86, 99] and a hyaluronidase, most likely GPI-anchored hyaluronidase 2 [100]. The presence of a protease, such as ADAMTS5 (aggrecanase) is likely also involved in order to remove associated proteoglycans (aggrecan and versican) [47]. CD44 rapidly transports (*t*_1/2_ of ~15 min) the fragmented HA (20–30 kDa) with any remaining bound proteins into an endosomal compartment distinct from coated pits and pinocytotic uptake pathways. The fragments are then transported to lysosomes for complete degradation (*t*_1/2_ of ~3 h) (Figure 4) [86, 99]. Therefore, distinct sites for biosynthesis and catabolism of HA on the surface of cells could effectively cooperate in controlling its dynamic metabolism. The stability of cytosolic HAS2 is significantly increased when serine 221 on Has2 is O-GlcNAcylated [86, 101]. Recent studies from our laboratory indicate that the matricellular protein periostin regulates HAS2 activation at a serine residue in embryonic heart valve remodelling [102]. It is possible that O-GlcNAcylation of this serine is a key for regulating whether or not HAS2 remains inactivated in response to periostin during development of the heart valve [102], which would allow the enzyme to migrate to the cell surface after its synthesis in the ER. There is increasing evidence that phosphorylation of serine and threonine residues in HAS2 to control hyaluronan synthesis whether or not it is activated [86, 103]. The phosphoserine increases when HA synthesis increases and phosphothreonine increases when HA synthesis decreases, as it is expected from the data discussed by Hascall's group [86].

HA, either alone or more often through its interaction with its binding partner CD44 on the cell membrane, is crucial for the tissue morphogenesis. For example, while the HAS1 and HAS3 null mice are developmentally normal, HAS2 deletion results in lethal defects in cardiac development and vascular abnormalities. TGF-*β*2-induced HAS2 expression and subsequent HA-CD44 signaling are required for endocardial cushion formation in HAS2-null mice [104–107]. Recent studies demonstrate that the balance of HA produced by distinct HAS enzymes is important for regulating inflammatory responses and wound contraction in the skin after injury [108]. At physiological pH, HA is a highly polyanionic molecule associated with counter ions, such as Na+, K+, Ca2+, and Mg2+. HA is characterized by its ability to occupy large hydrophilic solvent domains due to its very large size, which helps maintain the extracellular space and facilitates the transport of small molecular weight solutes through its domain. Solutions of high molecular mass HA exhibit time-dependent viscoelasticity because of polymer chain entanglement [109]. During rapid growth and tissue remodelling, the viscoelastic properties of HA depend on its molecular weight. The hydrated domain and the viscoelastic properties of HA are relevant for the application of HA in tissue repair as has been known for decades. In addition to the physiochemical effects of HA, HA also mediates the migration of fibroblasts to the wound site [110, 111].* In vitro* studies have demonstrated that, in the presence of specific growth factors, the higher the levels of HA, the greater the cell migration in cell cultures [14, 102, 112–116]. Most of the effects of HA upon cell behavior are mediated via interactions between HA and the HA receptors, CD44 [7, 14, 111, 113, 117–120] and RHAMM [121–124], through which intracellular signalling pathways are activated.

In skin wound healing, the differentiation of fibroblasts to myofibroblasts is very important for the closure of wounds and for the formation of the collagen-rich scar. In this regard, various studies have pointed to an important role of HA and HAS enzymes in regulating fibroblast-to-myofibroblast conversion. Work by the group of Steadman and Phillips has shown that the pericellular HA coat that surrounds human dermal fibroblasts appears to regulate profibrotic behavior of these fibroblasts, such that inhibition of HA synthesis significantly reduces TGF-*β*1-driven fibroblast proliferation [125] and transformation to myofibroblasts [126]. Furthermore, the mechanism by which HA regulates TGF-*β* signaling effects in the fibroblasts appears to involve changes in colocalization of the HA receptor (CD44) and the epidermal growth factor receptor (EGFR), both of which interact in the plasma membrane within lipid rafts [127–129]. Strong evidence for an important link between HA, CD44, and fibrotic processes is also found in the lung (as discussed later in Section 4.2.1). At another level, HA in the skin appears to regulate cytokine production and secretion in healing wounds by regulating the influx of leukocytes into the wound area. For example, selective loss of Has1 and Has3 (in Has1/Has3 double knockout mice) leads to a proinflammatory milieu that favors recruitment of neutrophils and macrophages in the connective tissue (dermis) [108]. In the Has1/Has3 double knockout mice, the rate of wound closure is accelerated (rather than inhibited), despite loss of HA-synthetic capacity in the skin epithelium and a reduction in overall HA levels in the dermis [108]. One possible explanation for this rapid wound closure is the observation that neutrophils and macrophages are recruited in greater numbers from small cutaneous vessels at the wound sites [108]. The abundant leukocytes secrete higher amounts of cytokines (e.g., TGF-*β*1), which probably activate local fibroblasts, making them more contractile and promoting their transformation into myofibroblasts, which thereby contracts the wounded [108]. The mechanism for robust neutrophil/macrophage recruitment in the Has1/Has3 mice is currently unknown. In a third example of how HA is important in fibrosis, overactive fibroblast behavior contributes to the pathogenesis of progressive fibrotic disorders such as scleroderma [71, 130–132]. Recent studies have shown that a critical element in the etiology of scleroderma is the presence of abnormal paracrine signaling involving signal-amplification loops between skin fibroblasts and the overlying keratinocytes. When keratinocytes from scleroderma patient skin are cocultured with fibroblasts, the fibroblasts were stimulated to produce more ECM due to dysregulated paracrine signaling involving IL-1 and TGF-*β* [130]. Given the importance of HA in regulating fibroblast responses to TGF-*β* and other cytokines, the potential for involvement of HA and CD44 in fibrotic processes of the skin appears to be ripe for future investigation.

In the lung, Has-mediated HA synthesis also has a vital role in repair after tissue injury. In the human disease* idiopathic pulmonary arterial hypertension*, increased HAS1 and decreased HAS2 levels are observed in pulmonary artery smooth muscle cells isolated from the patients, in whom total lung HA concentrations are also increased [82]. In a mouse model of* asthma*, expression of HAS1 and HAS2 is increased in lung tissue [133]. Conditional deletion of HAS2 in mesenchymal cells in *α*-smooth muscle actin (*α*-SMA)-HAS2 transgenic mice abrogated the invasive fibroblast phenotype, impeded myofibroblast accumulation, and inhibited the development of lung fibrosis [83].

#### 3.4.4. HA Degradation

High molecular weight HA has many crucial structural and physiological functions in wound repair following injury on the basis of its molecular weight and accessibility to various HA-binding proteins (HABPs) [67]. HA synthesis and degradation are tightly regulated during embryonic development and homeostatic processes. HA is removed from the ECM as a consequence of local catabolism. In mammals, the enzymatic degradation of HA results from the action of 5 functional hyaluronidases (Hyals), of which Hyal1 and Hyal2 are the two most common and ubiquitously important [71]. Hyal1 and Hyal2 are considered to be the main active HAases in tissues in almost all somatic tissues [134]. No HAase activity for human Hyal3 has been shown [127], and, in mice, Hyal3 does not seem to have a major role in constitutive HA degradation [135]. Recently, a novel HAase (KIAA1199) has been described, which is also detectable in human skin [136]. The larger isoform of Hyal1 is often secreted by the cell, while the smaller isoform is retained in acidic intracellular vesicles and lysosomes [137]. Hyal2 is often found as a glycosylphosphatidylinositol- (GPI-) anchored form, tethered to the extracellular side of the plasma membrane [138, 139]. By catalyzing the hydrolysis of HA, a major constituent of the interstitial barrier, Hyals lower the viscosity of HA, thereby increasing tissue permeability. Hyal1 has the maximal HA-degrading activity at pH 3.5–3.8 and cleaves HA to small oligosaccharides, which is consistent with its role of activity within lysosomes [140]. Hyal2 shows optimal activity at pH 6.0-7.0 and cleaves high molecular weight HA into intermediate size fragments of ~20 kDa [127].

HA degradation products stimulate endothelial cell proliferation, migration, and tube formation following activation of specific HA receptors, in particular CD44 and RHAMM [141]. HA fragments are implicated in the progression of lung diseases [142], and Hyals are elevated in scleroderma, a fibrotic lung disease [138, 143]. Furthermore, reactive oxygen species (ROS) can fragment HA under oxidative conditions [144]. HA catabolism by Hyals and ROS creates products that have biological activities distinct from native high molecular weight HA. HA fragments less than 20 disaccharides have been shown to be angiogenic [145]. Low and intermediate molecular weight HA (2 × 10^4^–4.5 × 10^5^ Da) can stimulate gene expression in macrophages, endothelial cells, eosinophils, and certain epithelial cells [146–149]. HA at ~200 kDa represents an interesting therapeutic strategy as it promotes reconstruction of a functional epithelium monolayer* in vitro* [150]. On the other hand, excessive HA degradation products also promote fibrotic scar tissue formation [151, 152].

#### 3.4.5. HA Receptor Interaction Induces Signaling in Wound Healing and Fibrosis

HA is involved in embryogenesis, wound repair, and tissue regeneration [142]. Skin wounds on early mammalian embryos heal perfectly with no signs of scarring and with complete restitution of the normal skin architecture [153], and the wound fluid HA is of high molecular weight [154]. During tissue injury and inflammation, HA that is normally present as high molecular weight (>1000 kDa) is modified into monocyte-adhesive matrices that stimulate immune cells at the injury site to express inflammatory genes through interactions with cell surface receptors. This leads to the release of enzymes and free radicals, which break the long chain molecules to lower molecular weight forms that have extraordinarily wide-ranging and often opposing biological functions, owing to the activation of different signal transduction pathways [155]. Studies have shown that HA fragments of lower molecular weight (~50–200 kDa) are proinflammatory, immunostimulatory, and proangiogenic, and they competitively bind to HA receptors on cell surfaces [156] (Figure 4).

While HA fragments may be important in initiating the inflammatory response, removal of these fragments is also critical for the resolution of the repair process [157]. Initial studies indicated that signaling initiated by HA degradation products involves CD44 primarily. However, studies of CD44-null macrophages indicate that there are other signaling pathways, notably through toll-like receptors, TL-R2 and TL-R4 [85]. Biological functions of HA and HA fragments are manifested through its interactions with a large number of HA-binding proteins (HABPs or hyaladherins) that exhibit significant differences in their tissue expression, specificity, affinity, and regulation [4, 7, 84, 118, 158–163]. A number of HABP bind HA through binding motifs, known as the link module, which consists of a span of ~100 amino acids that binds HA when oriented in the correct tertiary structure [164]. HABPs are constituents of the ECM, stabilize its integrity, and are involved in cellular signal transduction dependent on the molecular weight of HA and the cell phenotype [165].

Generally, HABPs interact with a minimum of 6–10 sugar residues of HA [142]. Therefore, a single chain of high molecular weight HA can theoretically accommodate on the order of 1000 HABPs [166]. The HABP link module family includes the link proteins, the PGs aggrecan, versican, brevican and neurocan, CD44 standard and variants, tumor necrosis factor-*α* stimulated gene 6 (TSG-6), and lymphatic vessel endothelial receptor 1 (LYVE-1) [167–169]. Studies have shown that, in response to HA of 40–400 kDa, the NF-*κ*B-mediated gene expression is activated by HA binding with HA receptor for endocytosis (HARE) [170]. The RHAMM receptor is an unrelated HA-binding protein with a HA-binding site peptide motif (B(X7) B) of minimal size of interaction with HA. CD44 and RHAMM are well-studied receptors associated with tissue injury, repair, cancer cell growth, and metastasis [4, 14, 159, 163, 171–173]. In addition, the binding of HA to intracellular adhesion molecule (ICAM-1) may affect its binding to other receptors at early stages of inflammatory activation [174].

#### 3.4.6. CD44 in Wound Healing and Fibrosis

**Figure 5:**
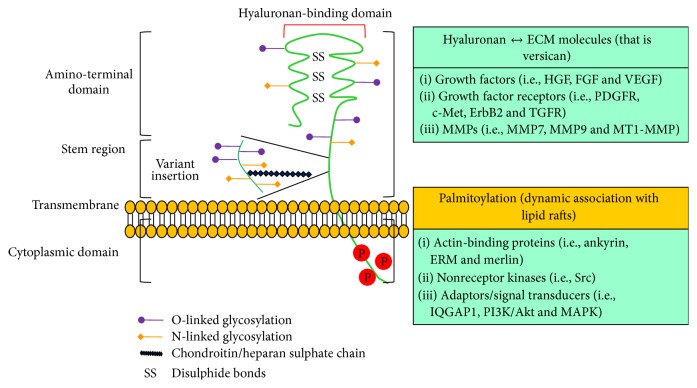
Structure, binding domains, and interactions of CD44 (Adapted from [7]). The ectodomain of CD44 contains HA-binding motifs and can contain chondroitin sulfate or heparan sulfate chains that can affect its HA-binding capacity and enable its interactions with growth factors and growth factor receptors, and its interaction with matrix metalloproteinases (MMPs). Transmembrane and cytoplasmic domains undergo multiple posttranslational modifications, including palmitoylation and phosphorylation on cysteine and serine residues, respectively, promoting the binding of proteins with crucial functions in cytoskeletal organization and signaling. ErbB2: epidermal growth factor receptor-2; ERM: ezrin–radixin–moesin; FGF: fibroblast growth factor; HGF: hepatocyte growth factor; IQGAP1: IQ motif containing GTPase activating protein 1; MAPK: mitogen-activated protein kinase; PDGFR: platelet-derived growth factor receptor; PI3K: phosphoinositide 3-kinase; TGFR: transforming growth factor receptor; VEGF: vascular endothelial growth factor.

The constitutive expression of CD44 and HA by a wide variety of cells implies that the interaction between these molecules is regulated. CD44 is the best characterized transmembrane HA receptor and because of its wide distribution it is considered to be the major HA receptor on most cell types [169]. CD44 is a structurally variable and multifunctional cell surface glycoprotein encoded by a single gene [175] (Figure 5). The genomic structure of CD44 consists of 21 exons [175] and the CD44 gene expression varies in size due to insertion of alternatively spliced variable exons derived from exon6–exon14 to form CD44v1–CD44v10 that are located in the membrane-proximal extracellular CD44 domains [176], approximately where N-terminal sequence homology between CD44 molecules from different species ends. The standard CD44 (CD44s) has a molecular weight ~90 kDa and exhibits extensive N-linked and O-linked glycosylation of the extracellular region, emphasizing the glycoprotein nature of CD44. CD44 can be induced to bind HA in cells activated with inflammatory stimuli, including cytokines, such as TNF-*α*, IL-*α*, IL-1*β*, IL-3, granulocyte-macrophage colony stimulating factor (GM-CSH), and interferon-*γ* (IFN*γ*) [84, 177, 178]. The molecular mechanisms underlying the induction of CD44-mediated HA binding include increased expression, variable glycosylation, receptor clustering, GAG attachment, phosphorylation, and inclusion of variant exons in the receptor [6, 7, 177, 179–184].

The bioactivity of the HA fragments strongly depends on their molecular weight. We and others have shown that malignant cells produce HA in order to activate their tumorigenic functions [7, 113, 117, 119, 120, 182, 185–190], while smaller oligosaccharides (~2-3 kDa) can ameliorate these effects* in vitro* [191, 192]. The variant 6 isoform, CD44v6, is of particular interest because it is overexpressed in many cancers, and HA-CD44v6 promotes growth [6, 7, 118, 193–198], which has a significant role in disease onset and progression. An increase in serum soluble CD44v6 due to MMP cleavage, along with serum HGF and HA levels, may serve as companion biomarkers for the presence of tumors and their responsiveness to CD44v6 [199–205]. We have shown that HA-CD44v6 signaling promotes tumor cell survival and tumor growth [182]. In addition, HA binding to CD44v6 is more avid than to CD44s and results in altered signaling [206–208]. In addition, CD44v6 mediates cross-talk between CD44v6 and receptor tyrosine kinases (RTKs), including c-Met [14, 209]. We also demonstrated that periostin, a fibrogenic matricellular protein, also activates HAS2 thus releasing free HA [102], which interacts with CD44 to regulate phenotypic transitions of lung fibroblasts to an invasive “myofibroblastic” phenotype, characterized by the overexpression of CD44, collagen 1, and *α*-SMA [14] (Figure 1). Overexpression of HAS2 by *α*-SMA positive myofibroblasts produced fatal lung fibrosis, whereas conditional knockout of HAS2 in myofibroblasts reduced the development of lung fibrosis. Moreover, CD44 contributed to the progressive fibrotic phenotype because lung fibrosis was reduced by either crossing the *α*-SMA-HAS2 transgenic mouse with the CD44 deficient mouse or by treatment with a blocking antibody to CD44.

All the functional effects of HA in inflammation and fibrosis may not be mediated by CD44. The role of CD44 in HA binding and signaling has recently been investigated in hematopoietic cells from CD44-null mice [210]. CD44-null mice develop normally and exhibit minor abnormalities in hematopoiesis and lymphocyte recirculation [210], indicating that the lack of CD44 can be compensated for in CD44-null mice. The induction of inflammatory gene expression in response to HA was observed in the CD44-null bone marrow cultures and in dendritic cells. It has been shown in wound healing or tissue injury that there is a potent mechanism for clearing HA following the injury. However, CD44-null mice challenged with bleomycin in an experimental model of lung injury accumulate extensive HA matrix that is not removed by the recruited macrophages with the resulting compromise of oxygen exchange, which results in death [83]. This suggests that CD44 may have evolved as a defence mechanism required for survival. Studies with the CD44-null mice and tissues have discovered the differential effects of CD44 in the predominant cell types that mediate host injury, suggesting potential roles for CD44 in mediating pathogenesis of host injury [172, 211]. For example, administration of IL-2 to wild-type mice triggered a significant vascular leak syndrome (VLS) in the lungs and liver. In contrast, in CD44-null mice, VLS induced by IL-2 was markedly reduced in the lungs and liver [211]. CD44-null mice exhibit enhanced hepatitis in a ConA-induced hepatocellular injury model [212]. Future studies in CD44-null mice will elucidate the importance of HA homeostasis and provide new insights into the role of CD44* in vivo* and in the tissue/cell models required to study the mechanisms of action of CD44 at the cellular and molecular levels of tissue injury and repair.

## 4. Therapeutic Approaches Relevant to Hyaluronan and CD44 in Wound Healing and Fibrosis

### 4.1. Therapeutic Approaches for Wound Healing

#### 4.1.1. Exogenous Application of Hyaluronan in Wound Healing

The viscoelastic and hydrated domain properties of HA are relevant for exogenous application of HA in tissue repair and regeneration processes. Exogenous application of HA accelerates skin wound healing in various animal models, including rats and hamsters [213–215]. Corneal epithelial wound healing by exogenously applied HA has been known for decades [216]. Laurent et al. [217] showed that exogenous HA can promote scarless healing in tympanic membranes, and Balasz and Denlinger [218] hypothesized that HA rich matrices can inhibit fibrous scars. Later, it was shown that* in utero* scarless fetal tissue repair is associated with high overall levels of HA for longer periods, indicating that high levels of HA may in part reduce collagen matrix deposition and contribute to scarless tissue repair [219]. In the older (late gestation) fetus and in adults, a reduction in HA levels is associated with fibrotic scarring [151]. Mack et al. demonstrated scarless healing in adults in an animal model, Hoxb13 knockout mice, in which HA levels remain elevated in adult skin [220]. Conversely, when Hoxb13 is overexpressed in the epidermis, HA levels are suppressed* in vitro* [221] and the skin behaves as a profibrotic wound-healing environment* in vivo* [222]. Although older findings in the literature regarding HA levels and wound healing were rather difficult to interpret in the past, newer ideas about the role of HA in regulating cytokine receptor signaling at the individual target cell level may help to reconcile the role of HA in fibrosis and healing in the future, as discussed further below.

#### 4.1.2. Proteins Associated with Hyaluronan Are Critical Determinants of Tissue Remodeling [223]

The effects of different HA preparations in the following studies are attributed to differences in growth factor and cytokine presentation to HA, and to HA receptor mediated molecular organization. The identification of the biological activities of various growth factors and cytokines in wound healing suggest that cells in injury models can respond to peptide factors for the long-term repair processes. Several growth factors derived from fibroblasts affect HA production [224]. For example, fibroblasts derived TGF-*β*1, b-FGF, PDGF, and EGF stimulate HA synthesis synergistically, and their effects on cell proliferation are through HA-initiated pathways, indicating the benefits of exogenous application of HA on ECM remodelling. It has been shown that fetal and adult fibroblast cells react differently to HA [151]. The former produce more ECM protein when HA is added to the culture, show greater migration to HA* in vitro*, and are insensitive to the applied PDGF, b-FGF, and EGF [225]. PDGF induces expression of TGF-*β*1 in adult wounds, which suggests that some of the longer term effects of PDGF are achieved indirectly by activation of TGF-*β*1 by fibroblasts within granulation tissue [226]. Clinical studies have also demonstrated that exogenous application of PDGF-AB together with other growth factors to chronic wounds can accelerate their closure [227, 228]. Application of EGF to organotypic cultures of epidermal cells leads to increased HAS with increased proliferation and migration, and TGF-*β*1 inhibits this response, a finding that shows why scarring wounds heal slowly [95]. Fetal and adult wound healing also differ with respect to the participation of various cytokines, particularly members of the TGF-*β* family. Increased canonical Wnt signaling occurs during postnatal wound repair but not during fetal cutaneous wound repair [229]. In this regard, TGF-*β*1 and TGF-*β*2 have been detected in adult wounds, while TGF-*β*3 is the principal isoform found in fetal wounds in response to rWnt3a protein [229]. Moreover, increased levels of macromolecular HA lead to decreased scarring in fetal life, whereas adult fibroblasts increased scarring due to increased HA breakdown products [230]. In addition, rapid wound closure is reported in HAS1/HAS3 double knockout mice, which have decreased amounts of HA in the skin, and wounding is accompanied by increased efflux of neutrophils into the tissue and by an earlier onset of myofibroblast differentiation [108]. In this case, increased inflammation might compensate for the decreased HA. Thus, in clinical settings, HA-protein (growth factor/cytokine) complexes may ameliorate the scarring [231]. It is likely that addition or removal of combinations of growth factors, or other agents such as protease inhibitors, will be more beneficial in some clinical circumstances.

We have shown that manipulating HA concentrations and HA-CD44 interactions can alter signaling pathways with many regulatory and adaptor molecules, including Src kinases, Rho-GTPases, PI3kinase, ankyrin, and ezrin [7, 118]. The engagement of CD44 with HA can modify cell survival and proliferation by changing intracellular engagement of ERM proteins [192, 232]. Additionally, HA may activate several receptor tyrosine kinases and HA-CD44 may promote clustering [233] and cooperate with other growth factor receptors [188, 190]. Additionally, we have shown that silencing variant 6 (CD44v6) inhibits tumor growth* in vitro* and* in vivo* [117, 182]. Moreover, blocking CD44v6 inhibits fibrogenesis of fibroblasts in scleroderma lung fibrosis [14], and blocking HA-CD44v6 downregulates fibroblast contractility [102]. Therefore, HA-CD44 variant interactions may modify several signaling pathways not directly related to CD44, but to other receptors that may interact with CD44 [234]. Thus, in clinical settings, increased HA in response to TNF-*α*, IL-*α*, IL-1*β*, IL-3, GM-CSH, and IFN*γ* [84, 177, 178] may promote HA-CD44 clustering and synergize with the cytokine receptor signaling pathways to induce fibrotic responses. This could explain how small HA fragments not capable of bridging receptors can alter these responses.

#### 4.1.3. ECM Remodeling by Manipulating the Interaction of HA with Other Matrix Molecules

Based on the studies above, there is now increasing evidence that HA can be used in biomedical applications for beneficial effects in wound healing. Bioengineered material used in research generally contains ECM molecules, including collagen and HA. The collagen matrix was developed by Bell et al. [235] and is commercially available as Apligraft. Later, a matrix containing collagen and CS has been available as Integra [236]. Coculture of Apligraft with neonatal foreskin fibroblasts and keratinocytes significantly alters the composition of the matrices produced [237]. The problem with these matrices is that the collagen used is xenogeneic bovine collagen.

Identification of HA for its ability to augment keratinocyte proliferation, fibroblast migration, and endothelial cell angiogenic responses in the wound bed makes it a useful biopolymer for wound healing, and pretreatment of HA matrices with fibroblasts has been applied to human wounds [238]. In contrast to collagen, HA is identical between species and has been used to make biomaterials by stable chemical modifications that have been used for wound healing. Furthermore, the degradation of HA matrix can have many effects on the regenerating wound, including water homeostasis, enhancement of angiogenesis, and collagen deposition and organization, which can benefit epithelial regeneration. HA also has free-radical scavenging properties. For example, benzyl esters of HA (HYAFF p80 and HYAFF p100), with differing degradation profiles, were used with a Laserskin method to treat both chronic and acute wounds, which showed excellent results in promoting angiogenesis in the wound bed and epithelial engraftment after 14 days and wound healing without contraction [239]. HA scaffolding material, including thiol-functionalized derivative HA-DTPH, has already been shown to be completely biocompatible in tissue engineering and implantation to provide three-dimensional templates that can improve cell growth and growth factor presentation [240–243]. Application of a cross-linked HA derivative (polyethylene glycol diacrylate- (PEGDA-) cross-linked HA-DTPH (HA-DTPH-PEGDA)) strongly inhibited contraction of a collagen matrix, whereas high molecular weight HA (HMW HA) facilitated collagen gel contraction. This suggests that manipulating the interaction of HA with other matrix molecules can alter ECM remodeling in wound healing [244]. HA is known to have a very short half-life of several hours in the body, which should be overcome for tissue augmentation applications. The residence time of HA can be prolonged by cross-linking HA in cosmetic fillers by the chemical modification of carboxyl groups of HA [245, 246] because they are in recognition sites of hyaluronidase (Hyal2) [247] and HA receptors [248, 249].

### 4.2. Therapeutic Approaches for Fibrosis

Fibrosis is the accumulation of ECM components in organs or tissues and is a fundamental feature of systemic sclerosis (SSc) [250, 251]. We are studying wound healing in SSc, which affects the skin and many internal organs, including the lungs, the gastrointestinal tract, and the heart. We will discuss a few therapeutic strategies and possible agents designed to inhibit pathologic mesenchymal phenotypes in SSc fibrosis, including treatment approaches that modulate inflammatory pathways, inhibit profibrotic growth factors, modulate epigenetic codes, and interfere with mesenchymal phenotype.

#### 4.2.1. Role of HA-CD44 Interaction on Profibrotic Growth Factors and Cytokines

As discussed in previous sections, many cytokines are involved in tissue repair, PDGF, EGF, FGF, and IGF1, and they can have many different roles in the healing process, ranging from regulation of cell proliferation, differentiation, and chemotaxis to directing wound remodeling by regulating ECM synthesis and degradation. These proteins may be locally synthesized and released as polypeptide growth factors and cytokines, which then have key roles in regulating cell and tissue functions.

Besides these, TGF-*β*1 is a fundamental component of tissue regeneration and repair. It increases profibrotic signals that promote biosynthesis of important components of the ECM, including collagens, CTGF, collagen receptor integrins, decorin, and TIMPs [252]. TGF-*β*1 is secreted at sites of injury by platelets and monocytes as well as by other cells, which promotes autocrine and paracrine interactions. We showed that TGF-*β*1 autocrine signaling in SSc fibroblasts induces a sustained expression of CD44v6, which interacts with HA and activates cell cycle progression and *α*-SMA production via Erk activation that increases collagen matrix synthesis. Inhibition of TGF-*β*1, or blocking CD44v6 by CD44v6siRNA, reduces these functions of SSc fibroblasts significantly [14]. We postulated that when TGF-*β*1 stimulation of fibroblasts is inappropriate, that is, too much TGF-*β*1 or heightened sensitivity to TGF-*β*1 due to autocrine signaling, pathologic fibrosis ensues with sustained HA-CD44v6 that will eventually overwhelm the system in favor of profibrotic effects [14]. In addition, we postulated that the increase in antifibrotic hepatocyte growth factor (HGF) expression at the onset of chronic injury may initially compensate and support a regenerative process [14, 253], whereas repetitive lung injury results in overexpression of TGF-*β*1 that leads to the profibrogenic effects. Therefore, the balance between TGF-*β*1 and HGF appears to have a critical role in determining whether the injured tissues undergo recovery or fibrogenesis [14]. Fresolimumab is a human monoclonal antibody that inactivates all forms of TGF-*β*. In Phase I trials, fresolimumab was safe and well tolerated in patients with primary focal segmental glomerulosclerosis, IPF, and renal cancer. Similarly, Phase II trials of a human monoclonal antibody for CTGF (FG 3019) for patients with liver fibrosis (due to chronic hepatitis B infection) and pulmonary fibrosis are promising. Imatinib mesylate, used for the treatment of chronic myelogenous leukemia (CML), blocks both profibrotic TGF-*β*1 signaling and suppresses activity of the PDGF receptor [254, 255]. In SSc, however, the results are still inconclusive.

#### 4.2.2. Role of HA-CD44 Interaction in Mesenchymal Cell Activation

The mechanisms that regulate fibroblast to myofibroblast differentiation in SSc remain poorly understood. As many profibrotic pathways are linked to TGF-*β*1 signaling, novel antifibrotic therapies that target other pathways may indirectly act via suppression of TGF-*β*1. For example, peroxisome proliferator-activated receptor-*γ* (PPAR-*γ*) can suppress TGF-*β*1-dependent cell activation and collagen production in fibroblasts and inhibit the development of fibrosis in murine models [256]. Recent studies also suggest that NADPH oxidase 4 (NOX4) is essential for TGF-*β*-induced differentiation of fibroblasts to myofibroblasts* in vitro* and for bleomycin-induced pulmonary fibrosis* in vivo* [257]. The development of small molecule inhibitors and/or other strategies targeting NOX4 or the use of PPAR-*γ* agonists may abrogate fibrosis through antifibrotic mechanisms. Studies suggest that the RhoA/ROCK pathway is a critical regulator of contractility of mesenchymal cells, including lung fibroblasts from SSc patients [258–260]. Fasudil, a small molecule inhibitor of ROCK, has recently been studied in US populations for other disease indications (https://www.clinicaltrials.gov/). It also reduces myofibroblast activation in lung fibrosis in an animal model [260], suggesting a potential use of this compound for treatment of fibrotic diseases.

Finally, the profibrotic pathway linked to TGF-*β*1 signaling may directly act through a profibrotic mechanism through the augmentation of a HA-CD44 pathway. For example, HA can promote a profibrogenic activity in fibroblasts cells, as shown by changes in cellular behavior due to HA-CD44 interaction that induces biological processes. When hyaluronan synthase 2 (HAS2) was transgenically overexpressed by myofibroblasts* in vivo*, a severe fibrotic phenotype followed bleomycin-induced lung injury, presumably due to HA-CD44 function [83]. Mesenchymal fibroblasts that are derived from HAS2-deficient mice, or are treated with a CD44 blocking antibody, fail to show the same degree of fibrogenic function as do wild-type mice [83]. Our recent study [14] showed that sustained CD44v6-induced signals regulate myofibroblast proliferation, activation, and matrix deposition in SSc fibroblasts in response to autocrine TGF-*β*1 stimulation. This indicates that tissue specific blocking of HA-CD44 signaling by silencing CD44 using specific siRNA can be a viable approach to attenuate profibrogenic functions.

## 5. Conclusion

Together these studies address components of wound healing processes and describe a number of different mechanisms that have been implicated in the pathogenesis of defective wound healing that leads to progressive fibrosis disorders. Concepts relating wound healing to fibrogenic mechanisms have converged on a model of inflammation that coordinates with ECM components, soluble mediators that induce wound healing, and failure of tissue regeneration leading to fibrosis. HA-based novel therapeutic mechanisms that can use HA-biomaterials and antagonists to HA-CD44 signaling pathways are beginning to produce promising results in* in vitro* and* in vivo* models of both wound healing and fibrosis. Considering that promising studies sometimes do not translate into patient benefit under different biological conditions and disease states, care must be taken to ensure the long-term safety of using advanced engineering strategies and well-conducted and controlled clinical trials need to be evaluated before the therapeutic agents, or HA-based biomaterials can be recommended for defective wound healing. Our future studies will focus on determining the mechanisms by which HA-CD44 regulates impaired wound healing, with particular emphasis on microRNAs that regulate HA synthesis and CD44 biology in normal and pathological wound healing.

## Abbreviations

GAG: Glycosaminoglycan
PG: Proteoglycan
HA: Hyaluronan
ECM: Extracellular matrix
HAS: Hyaluronan synthase
Hyals: Hyaluronidases.
